# Seeds in Chernobyl: the database on proteome response on radioactive environment

**DOI:** 10.3389/fpls.2012.00231

**Published:** 2012-10-10

**Authors:** Katarína Klubicová, Martin Vesel, Namik M. Rashydov, Martin Hajduch

**Affiliations:** ^1^Department of Reproduction and Developmental Biology, Institute of Plant Genetics and Biotechnology, Slovak Academy of SciencesNitra, Slovakia; ^2^Emsy LtdBratislava, Slovakia; ^3^Department of Biophysics and Radiobiology, Institute of Cell Biology and Genetic EngineeringKyiv, Ukraine; ^4^Institute of Chemistry, Centre of Excellence for White-Green Biotechnology, Slovak Academy of SciencesNitra, Slovakia

**Keywords:** Chernobyl, radioactivity, plants, seeds, proteome, database

## Abstract

Two serious nuclear accidents during the last quarter century (Chernobyl, 1986 and Fukushima, 2011) contaminated large agricultural areas with radioactivity. The database “Seeds in Chernobyl” (http://www.chernobylproteomics.sav.sk) contains the information about the abundances of hundreds of proteins from on-going investigation of mature and developing seed harvested from plants grown in radioactive Chernobyl area. This database provides a useful source of information concerning the response of the seed proteome to permanently increased level of ionizing radiation in a user-friendly format.

## INTRODUCTION

Two large nuclear accidents in Chernobyl (1986) and Fukushima (2011) contaminated large agricultural areas with radioactivity. Surprisingly, plants grow and successfully reproduce in radio-contaminated areas. This unanticipated ability of plants to adapt to a contaminated environment is not well understood. Partial explanations can be found in the results from preliminary genomic and proteomic analyses. In wheat, there was an increase in the frequency of heterozygous structural variants for 13 single-copy monomorphic microsatellite loci (Kovalchuk et al., 2000), including gain and loss of repeats and the complete loss of microsatellite bands (Kovalchuk et al., 2003). Additionally, analysis of genomic DNA from pine trees growing in the radioactive area showed a threefold increase in mutations (Kuchma et al., 2011), and a substantial increase in hypermethylation (Kovalchuk et al., 2003). The progeny of *Arabidopsis thaliana* plants grown in the Chernobyl area were resistant to higher levels of mutagens than controls (Kovalchuk et al., 2003). These plants exhibited a 10-fold decrease in the frequency of extrachromosomal homologous recombination, an increased level in global genome methylation, and altered expression of selected genes (Kovalchuk et al., 2004). Despite the likelihood of significant changes in DNA in response to growth in a radioactive environment, only minor changes have been seen in the abundance of mature seed proteins. Results from comparative proteomic analyses of mature seeds grown in either radioactive or non-radioactive soil in the Chernobyl area revealed significant alterations in the abundance of only a small fraction of the complete seed proteome (Danchenko et al., 2009; Klubicová et al., 2010).

The database “Seeds in Chernobyl” (http://www.chernobylproteomics.sav.sk) was established in order to disseminate data from ongoing investigations of seed proteomes of plants grown in the radioactive Chernobyl area. Since 2007, soybean and flax seeds of local varieties are cultivated in radioactive and non-radioactive experimental fields established in the Chernobyl area (Danchenko et al., 2009; Klubicová et al., 2010, 2012). Seeds are harvested on a yearly basis, analyzed, and sown in order to obtain next generation seeds. To date, protein abundances in mature and developing seeds of two subsequent soybean generations and one generation of mature flax seeds are available. These analyses revealed, for instance, that increased heavy metals resistance and mobilization of seed storage protein are part of soybean growth in the radioactive Chernobyl area in two subsequent generations (Danchenko et al., 2009; Klubicová et al., 2012). Such adaptation also includes adjustments to carbon metabolism in the cytoplasm and plastids, increased activity of the tricarboxylic acid cycle, and decreased condensation of malonyl-acyl carrier protein during fatty acid biosynthesis (Klubicová et al., 2012).

The objective of this research is to enhance our understanding of the molecular processes employed during the plant adaptation to increased levels of ionizing radiation. This is important in order to develop strategies for agricultural recovery of areas contaminated with radioactivity for non-food purposes (Klubicová et al., 2011a,b), and to develop plant cultivation strategies that will be resistant to cosmic radiation for long space missions in the future.

## DATABASE STRUCTURE

To date, Seeds in the Chernobyl web-based database contain the data of protein abundances in soybean and flax seeds harvested from radioactive Chernobyl area. The database was designed for a Linux/Apache/MySQL environment using PHP 5 for HTML generation. A PHPExcel library was used for data reading. Protein abundance profiles were created using PChart library. The database structure is presently divided into three main areas: Soybean, Flax, and About the project. This structure will expand as the data from other crops will be released. The Soybean section is further divided into First and Second generations. The Flax section contains information from the first generation. These sections are independent, to allow for future updates.

The data of protein abundances are available through interactive 2-DE gel maps, where quantified and identified protein spots are highlighted. By clicking on a 2-DE spot, quantitative data is displayed. Alternatively, after the selection of protein from the protein table, the corresponding protein spot is highlighted in the 2-DE map with the additional display of associated quantitative data.

## FIRST GENERATION OF SOYBEAN SEEDS HARVESTED FROM THE CHERNOBYL AREA (SOYBEAN/FIRST GENERATION)

**FIGURE 1:**
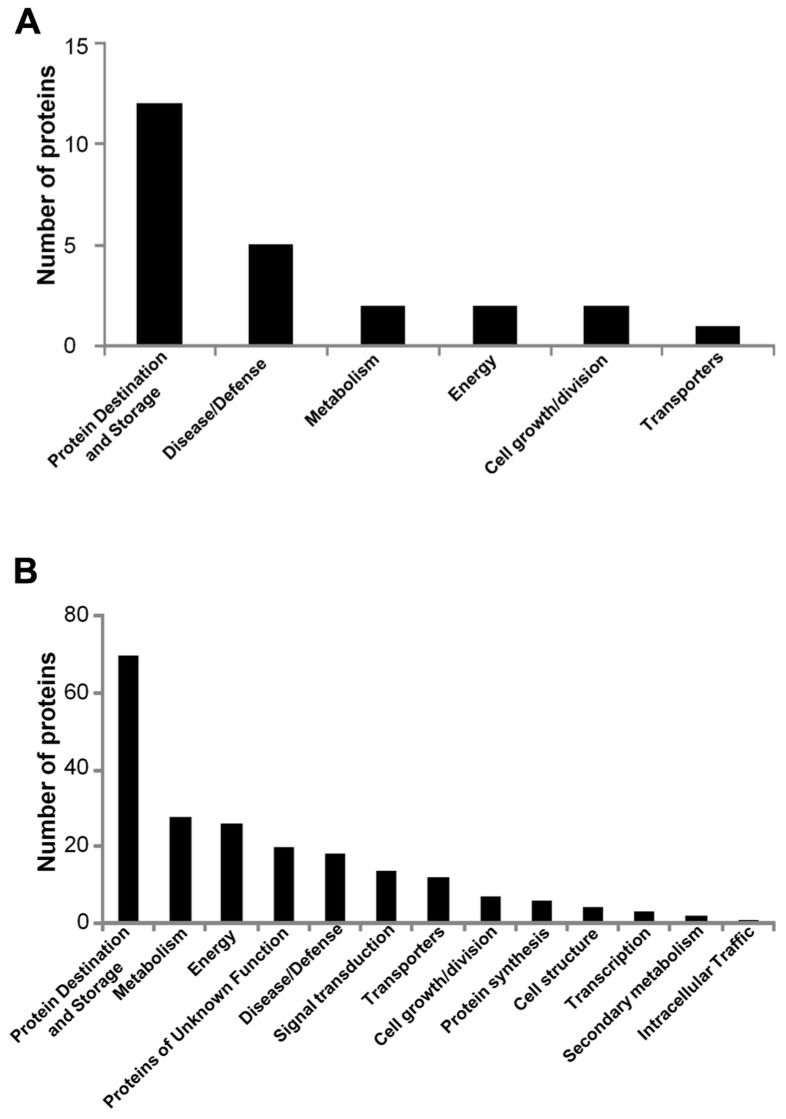
**Functional classification of soybean proteins contained in the database**. **(A)** The classification of 24 differentially abundant proteins detected on 2-DE gels of p*I* 4–7 between mature seeds of first generation harvested from non-radioactive and radio-contaminated Chernobyl area. **(B)** The 211 proteins quantified during seed development in second generation soybean plants grown in non-radioactive and radioactive Chernobyl areas.

The aim of this experiment was to identify differentially abundant proteins between mature seeds harvested from the first generation of soybean plants grown in non-radioactive and radioactive Chernobyl experimental fields. A total of 698 protein spots were quantified (Danchenko et al., 2009). However, only 64 of these spots could be considered to be differentially abundant when a *p*-value of ≤0.05 was imposed (Danchenko et al., 2009). In total, 26 of these proteins were identified using tandem mass spectrometry (MS/MS). The “Seeds in Chernobyl” database contains quantitative data for 24 of these proteins detected on 2-DE gels of p*I* range 4–7 (**Figure 1A**). This dataset contains 12 seed storage proteins, five proteins associated with disease/defense; two metabolic proteins, five proteins associated with energy, two proteins associated with cell growth/division, and one transporter (**Figure 1A**).

## SECOND GENERATION OF SOYBEAN SEEDS HARVESTED FROM THE CHERNOBYL AREA (SOYBEAN/SECOND GENERATION)

The aim of this experiment was to elucidate protein abundances during seed development of second generation soybean grown in the radioactive Chernobyl area. The abundance of 211 proteins was determined (Klubicová et al., 2012). This dataset contain 70 proteins associated with protein destination, 28 protein associated with metabolism, 26 energy proteins, 21 proteins of unknown function, 18 proteins associated with disease/defense, 14 signal transduction proteins, 11 transporters, and 7 proteins associated with cell growth/division (**Figure 1B**). The dataset contains protein abundances during seed development in non-radioactive and radioactive Chernobyl area (**Figure 1B**).

## FIRST GENERATION OF FLAX SEEDS HARVESTED FROM THE CHERNOBYL AREA (FLAX/FIRST GENERATION)

**FIGURE 2:**
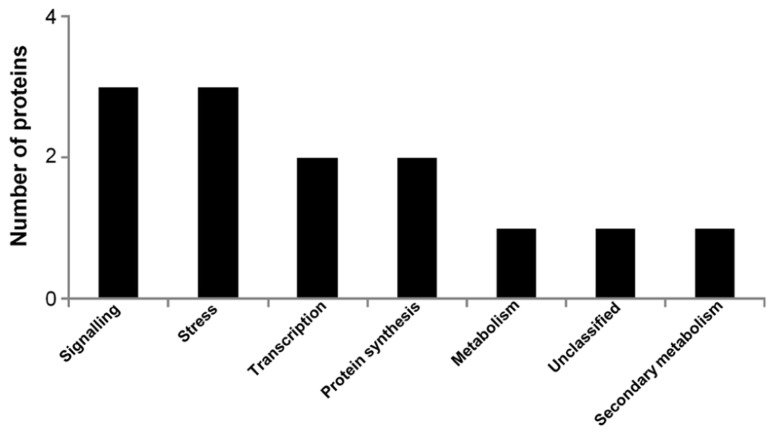
**Functional classification of 13 differentially abundant proteins detected on 2-DE gels of p*I* 4–7 between mature flax seeds of first generation harvested from non-radioactive and radio-contaminated Chernobyl area**.

The aim of this experiment was to identify differentially abundant proteins between mature flax seeds of first generation harvested from plants grown in Chernobyl experimental fields. In total, 720 protein spots were quantified and only 35 were differentially abundant when a *p*-value of ≤0.05 was used, out of which 28 were identified using MS/MS (Klubicová et al., 2010). Presently, the database contains the identity of 13 of these proteins that were detected on 2-DE gels of p*I* 4–7. The dataset contains three proteins associated with signaling, three proteins associated with stress responses, two transcription factors, two elongation factors, one metabolic protein, one unclassified protein, and one protein associated with secondary metabolism (**Figure 2**).

## FUTURE PERSPECTIVE

The Seeds in Chernobyl web-based database will be continuously updated with new data based on the progress of our research. It is expected that the database will contain the data of protein abundances from five generations of flax and soybean. Additionally, data from similar investigations with other plant species will also be deposited in this database.

## Conflict of Interest Statement

The authors declare that the research was conducted in the absence of any commercial or financial relationships that could be construed as a potential conflict of interest.
